# Mapping heat stress-induced core histone post-translational modifications in *Acropora cervicornis*

**DOI:** 10.1093/eep/dvaf017

**Published:** 2025-05-29

**Authors:** Cassandra N Fuller, Sabrina Mansoor, Santiago J Guzman, Lilian Valadares Tose, Serena Hackerott, Javier Rodriguez-Casariego, Jose M Eirin-Lopez, Francisco Fernandez-Lima

**Affiliations:** Department of Chemistry and Biochemistry, Florida International University, Miami, FL 33199, United States; Environmental Epigenetics Laboratory, Institute of Environment, Florida International University, Miami, FL 33199, United States; Department of Chemistry and Biochemistry, Florida International University, Miami, FL 33199, United States; Department of Chemistry and Biochemistry, Florida International University, Miami, FL 33199, United States; Environmental Epigenetics Laboratory, Institute of Environment, Florida International University, Miami, FL 33199, United States; College of Earth, Ocean, and Environment, School of Marine Science and Policy, University of Delaware, Lewes, DE 19958, United States; Environmental Epigenetics Laboratory, Institute of Environment, Florida International University, Miami, FL 33199, United States; Department of Marine Biology and Ecology, Rosenstiel School, University of Miami, Miami, FL 33124, United States; Environmental Epigenetics Laboratory, Institute of Environment, Florida International University, Miami, FL 33199, United States; Department of Chemistry and Biochemistry, Florida International University, Miami, FL 33199, United States; Biomolecular Sciences Institute, Florida International University, Miami, FL 33199, United States

**Keywords:** post-translational modifications, H4 histone, nLC-TIMS-ddaPASEF-TOF MS/MS, staghorn coral, *Acropora cervicornis*, H2A histone, H2B histone

## Abstract

Histone post-translational modifications (PTMs) participate in the dynamic regulation of chromatin structure and function, through their chemical nature and specific location within the histone sequence. Alternative analytical approaches for histone PTM studies are required to facilitate the differentiation between ubiquitously present isomers and the detection of low-abundance PTMs Here, we report a high-sensitivity bottom-up method based on nano-liquid chromatography (nLC), trapped ion mobility spectrometry (TIMS), data-dependent acquisition (DDA), parallel accumulation-serial fragmentation (PASEF), and high-resolution time-of-flight tandem mass spectrometry (ToF-MS/MS) for the analysis of histone PTMs. This method was tested in a threatened coral species, the staghorn coral *Acropora cervicornis*, during an episode of acute thermal stress. The obtained results allowed for the identification of PTM changes in core histones involved in the coral’s heat response. Compared to traditional LC-MS/MS approaches, the incorporation of TIMS and ddaPASEF MS/MS resulted in a highly specific and sensitive method with a wide dynamic range (6 orders of magnitude). This depth of analysis allows for the simultaneous measurement of low-abundance PTM signatures relative to the unmodified form. An added advantage is the ability to mass- and mobility-isolate prior to peptide sequencing, resulting in higher confidence identification of epigenetic markers associated with heat stress in corals (e.g. increased H4 4–17 with 2ac and 3ac, and decreases in H4 4–17 K12ac, K16ac, H4 K20me_2_, and H2A K5ac, K7ac, K9ac, K12ac, K14ac, and K74ac).

## Graphical Abstract

**Graphical Abstract:**
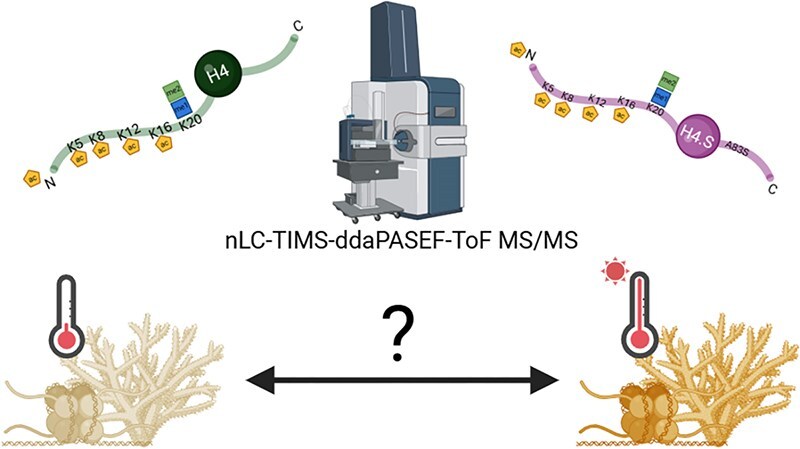

## Introduction

Histones are small basic proteins, which, contrary to a long-held belief, display a high level of diversity within the cell nucleus [1], including the ubiquitous coexistence of genetic variants and multiple proteoforms that bind to DNA and form the fundamental subunit of chromatin known as a nucleosome [2–5]. Within the nucleosome, dimers of the four core histones (H2A, H2B, H3, and H4) bind together to create the octameric face, from which the N- and C-terminal tails protrude [2–4, 6–8]. Due to this increased exposure, the highly basic N-terminal tails are especially amenable to interactions with the cellular machinery responsible for post-translational modifications (PTMs). These PTMs [e.g. acetylation (ac), methylation (me_1–3_), ubiquitination (ub), and phosphorylation (ph)] facilitate the modulation in the function of diverse genomic regions in response to abiotic or other biological signals [6, 9–11].

Traditionally monitored by antibody labelling [12–14], alternative analytical methods are required to account for the specificity needed to distinguish between isomeric histone proteoforms, particularly in the case of samples that antibodies remain unavailable for. Mass spectrometry (MS)-based proteomics methods are emerging and continuously evolving in terms of mass accuracy and resolution, which can provide a more comprehensive and cost-effective overview of the histone code [15]. Top-down MS proteomics provides annotation on intact proteins using ultrahigh-resolution MS to combat inherent chemical complexity and the biological diversity of histones [16–27]. We have demonstrated the advantages of dual gas-phase separation and complementary fragmentation techniques (e.g. top “double-down” MS), which aids in isomeric proteoform separation [20]. Another option is middle-down proteomics, which utilizes enzymes (e.g. chymotrypsin Glu-C, Asp-N) to cleave proteins into large peptides (>3 kDa) [28]. These methods, however, do not reliably account for the high isomeric content of histones due to cleavage being at the tail level [∼50 amino acids (AAs)], where a large majority of PTMs occur. To address this, gas-phase preseparation in combination with electron- or UV-based fragmentation can be employed for adequate peptide sequencing and PTM local elucidation [28–33].

The most common approach to MS proteomics is bottom-up. This method employs proteases (e.g. trypsin, Arg-C [34–36]) that cleave at high-frequency AAs (e.g. lysine and/or arginine in histones) and result in many small peptides (<3 kDa). Trypsin is commonly used due to its robustness and higher efficiency; however, since trypsin cleaves at both lysine and arginine residues, this digestion typically produces many peptides that are too short to yield confident sequence assignment in core histone analysis. To account for this, we have previously published both irreversible (e.g. propionylation) and reversible (e.g. citraconylation) derivatization methods that block lysine residues as cleavage sites [7, 37–41]. This facilitates the use of trypsin, which is more cost-effective and robust than Arg-C, and provides slightly longer peptides (still <3 kDa) for analysis. At this shorter peptide level, however, there is still a need to address isomeric content among peptides with multiple PTM sites [38, 41]. Recently, we sequenced and annotated *Acropora cervicornis* coral histone H4 proteoforms from a pooled sample set using top-down MS (https://doi.org/10.34703/gzx1-9v95/MM9SHA) [42]; however, appropriate methodology for diversity comparison between individual organisms has yet to be shown.

Here, we describe the analytical value of online nano-liquid chromatography (nLC), trapped ion mobility spectrometry (TIMS), data-dependent acquisition (DDA), parallel accumulation-serial fragmentation (PASEF), and high-resolution tandem time-of-flight (ToF-MS/MS) for the analysis of histone PTMs. With the recent reports of *A. cervicornis* (https://doi.org/10.34703/gzx1-9v95/MM9SHA) [42] histone H4, H2A, and H2B variants and sequences, we now applied bottom-up strategies to demonstrate the sensitivity and applicability of this method to heat stress-induced PTM changes.

## Materials and methods

### Sample preparation

*Acropora cervicornis* (staghorn) corals were obtained from the Coral Reef Foundation (CRF) nursery located at Tavernier, FL (N 24.982715°, W −80.436286°) and were propagated under CRF’s permit #FKNMS-2019-012-A2. Fragments represented clones of a single coral genotype (CRF AC112) to limit genetic variability during method development. Corals were separated into two temperature treatments including an ambient control (ctrl; *n* = 3) and heat-exposed (exp; *n* = 3) group. Corals in the ambient control treatment group were maintained at 26°C, while heat-exposed corals were subjected to acute (13 h) heat stress with a maximum temperature of 33°C with the aim of inducing a detectable molecular response to thermal stress.

Histones were extracted from collected fragments of each coral treatment group (∼10 cm each). Briefly, coral fragments were flash-frozen in liquid nitrogen and ground into a fine powder using a mortar and pestle. The calcium carbonate skeleton was removed from the coral tissue by suspending the powdered coral (∼250 mg/ml) in chilled 1X Pre-lysis buffer (EpiQuik™ Total Histone Extraction Kit, EpigenTek, Farmingdale, NY) on ice for 10 min to allow carbonate deposition [43]. The tissue slurry was carefully transferred to a Dounce homogenizer to continue the lysis and histone acid extraction using the EpiQuik™ Total Histone Extraction Kit. Acid-soluble proteins were subjected to acetone precipitation overnight as previously described [44]. The total protein concentration was determined using the Qubit Protein Assay Kit (Invitrogen, Carlsbad, CA). Isolated histones were resuspended in water and stored at −80°C until ready for further preparation steps.

The extracted histones were derivatized (propionylated, pr), as previously described [7, 37, 38, 40, 41, 45]. Briefly, histones were solubilized in 100 mM NH_4_CO_3_ (1 µg/µl, pH 8) and propionylation reagent [1:3 v/v propionic anhydride: acetonitrile (ACN)] was added (1:4 v/v), followed quickly by NH_4_OH (1:5 v/v) to maintain a pH of 8. The samples were incubated for 15 min, and then this procedure was repeated once before drying via vacuum centrifuge. Propionylated histones were reconstituted in NH_4_CO_3_ (1 µg/µl) and digested using trypsin (1:10 wt/wt) [40] overnight at room temperature. The digest was halted by freezing at −80°C for at least 1 h, and then samples were thawed and dried. The derivatization procedure was repeated as before to propionylate the newly formed peptide N-terminals. Fully propionylated peptide samples were desalted using homemade stage tips, as previously described [37, 38, 41]. The C_18_ tips were activated using ACN, followed by equilibration with 0.1% trifluoroacetic acid (TFA). Sample pH was reduced to 4 using glacial acetic acid, and then samples were loaded onto the C_18_ material and washed once with 0.1% TFA before elution using 0.5% acetic acid in 75% ACN. Desalted samples were dried, resuspended in 0.1% formic acid (FA), and spiked with custom histone-like QC peptides to monitor the reproducibility of the bottom-up MS method.

### Bottom-up histone PTM screening

A nanoElute 2 nLC system fitted with a C_18_ column (15 cm × 150 µm i.d., 1.5 µm, Bruker PepSeq column) kept at 50°C was coupled to a commercial timsTOF Pro2 mass spectrometer (Bruker Daltonics, Billerica, MA). The nLC separation gradient using 0.1% FA in water (mobile phase A) and 0.1% FA in ACN (mobile phase B) started at 2% B and continued as follows: (i) 0–60 min to 35% B, (ii) 60–69 min to 95% B, and (iii) 70–78 min to 2% B. Each injection consisted of 1 µl of sample containing 250 ng/µl propionylated coral histone peptides and 25 ng/µl spiked QC peptide (GVKFRGSTGGKAPRGKAPATSGMVGPHR, 2765.54 Da); the QC peptide can be traced using the final QC1 (pr-GSTGGK(pr)APR, 471.75^2+^) and QC2 (pr-GK(pr)APATSGMVGPHR, 739.38^2+^) propionylated digest targets. A CaptiveSpray nanoESI source (Bruker Daltonics) operated at 1200 V and flow rate of 500 nl/min was used as the nLC-MS interface. Tandem MS/MS was performed using collision induced dissociation (CID) on mobility and *m/z* selected precursor ions in ddaPASEF mode over a range of 0.60–1.80 1/K_0_ and 100–1700 *m/z* (1+ to 4+ charge states), respectively, while CID collision energy was stepped as a function of both *m/z* and 1/K_0_, as previously described [37, 41]. The limit of detection (LOD) is specific to each peptide target and charge state; to evaluate the fold changes, the LOD was estimated as the lowest value observed for a known peptide signal in a biological histone pulldown (see Fig. S7).

### Histone PTM data analysis

The bottom-up nLC-TIMS-PASEF-ToF MS/MS data were analysed using a custom script in DataAnalysis software (DA, v6.1, Bruker Daltonics) that considered *m/z*, mobility (1/K_0_), and retention time from a predetermined list of derivatized histone peptide sequences with varying PTMs (e.g. ac, me_1–3_). A target list based on H4, H2A, and H2B variants detected in *A. cervicornis* coral (H4, H4.S, H2A, H2A.A, H2B-1, H2B-2, H2B-2K, and H2B-3; https://doi.org/10.34703/gzx1-9v95/MM9SHA) [42] was used. The histone H3 sequence has not been reported and therefore was not included [42]. The QC1 and QC2 peptides were used as a measure of the analytical reproducibility and analysis performance. Mobility and mass method calibration was performed using hexakis(2,2-difluoroethoxy) phosphazine, hexakis(2,2,3,3-tetrafluoropropoxy) phosphazine, and hexakis(1H, 1H, 7H-dodecafluoroheptoxy) phosphazine standards (Apollo Scientific Ltd, UK). The reported peptides and PTMs were manually curated using the isotopic profile of the precursor mass (±0.01 Da), previously reported peptide mobility profile patterns (including positional isomers, relative standard deviation, RSD <2%)[41], and MS/MS fragmentation patterns. Extracted ion mass and mobility filtered chromatograms were extracted using the DA script and normalized to the area of QC peptides (e.g. QC1).

## Results and discussion

**Figure 1.**
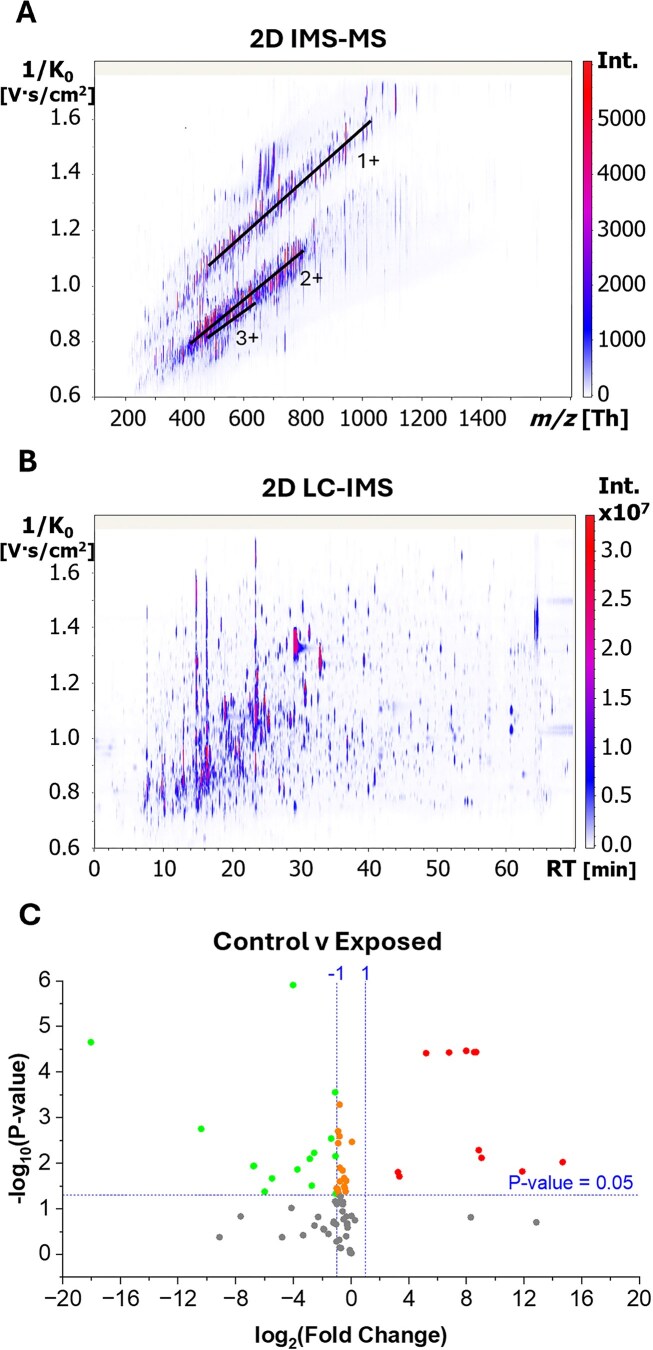
2D (A), IMS-MS, and (B) LC-IMS from nLC-TIMS-ddaPASEF-ToF MS/MS of bottom-up propionylated tryptic coral peptides and (C) volcano plot comparing histone PTM changes from control to exposed corals. The volcano plot was created by plotting the log2 of the fold change (*x*-axis, significance = *x* < −1 or *x* > 1) and −log10 of the 1:1 comparison *P*-values determined by two-tailed *t*-test (*y*-axis, 95% confidence interval) using the QC1-normalized areas.

The histone PTM screening using bottom-up nLC-TIMS-ddaPASEF-ToF MS/MS resulted in the detection of characteristic peptides and their identification based on retention time, mobility, and characteristic fragmentation pattern. Inspection of the 2D IMS-MS contour plot (Fig. 1A) shows distinct separation of the peptides based on their charge state; peptides were observed in the 1+ to 3+ charge state range, in good agreement with previous results as a consequence of the derivatization step (i.e. propionylation protocol neutralizes most basic AAs [7, 38, 41]). Inspection of the 2D LC-IMS contour plot shows the separation of most coeluting peptides, demonstrating the advantages of the added mobility separation prior to MS/MS (Fig. 1B).

**Figure 2.**
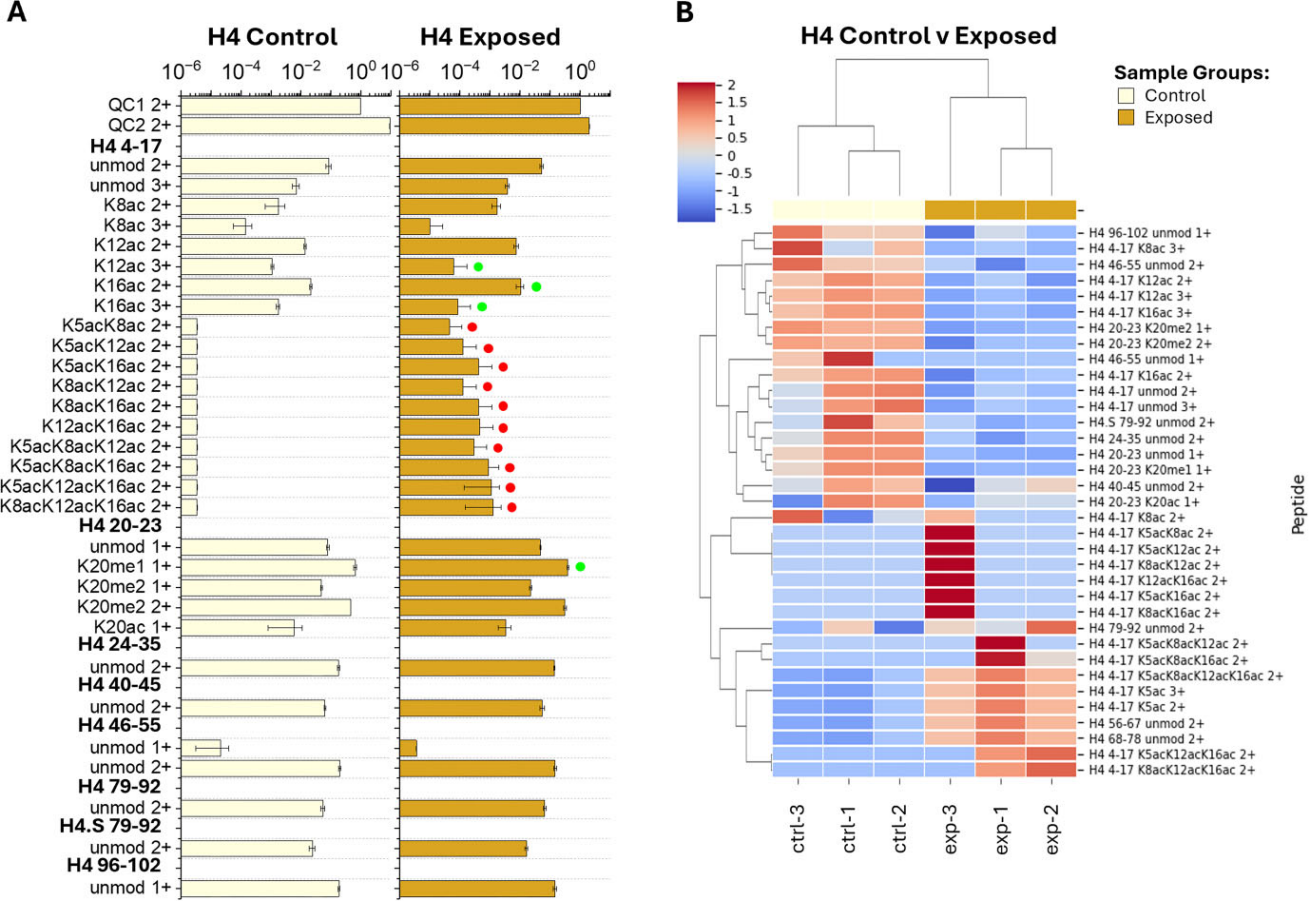
(A) Detected H4 bottom-up peptide areas normalized to QC1 from control and exposed coral samples with up- and downregulated peptide features distinguished by red and green dots, respectively. Bottom-up peptide feature bar plots shown were created from the QC1-normalized area means (bar) and standard deviations (error) across biological replicates. (B) Heatmap grouping samples (*x*-axis) by control (light yellow) and exposed (gold) corals and H4 peptides (*y*-axis) by *z*-score (complete comparison) of the QC1-normalized area values with dendrograms displayed using Euclidean distance.

**Figure 3.**
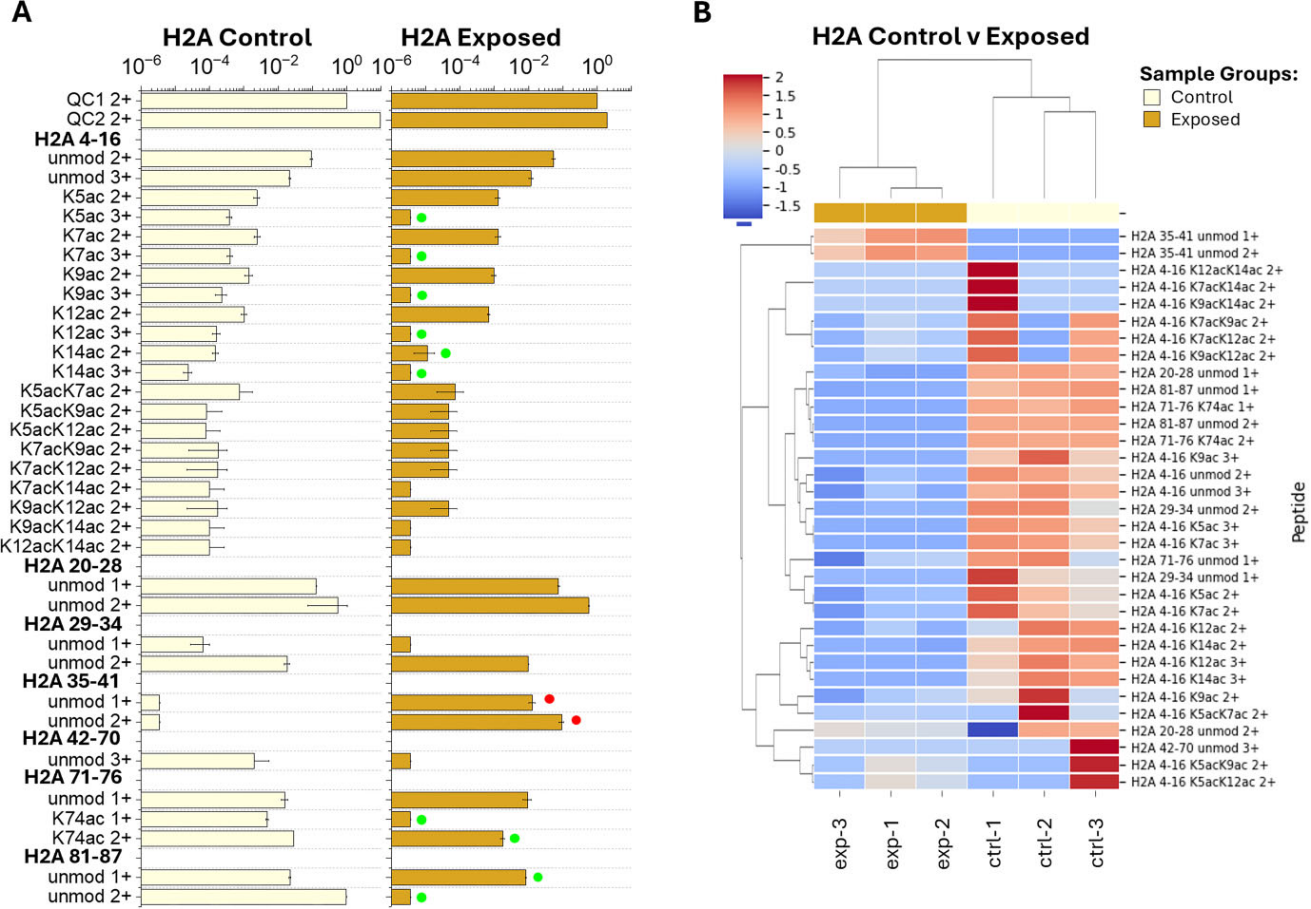
(A) Detected H2A bottom-up peptide areas normalized to QC1 from control and exposed coral samples with up- and downregulated peptide features distinguished by red and green dots, respectively. Bottom-up peptide feature bar plots shown were created from the QC1-normalized area means (bar) and standard deviations (error) across biological replicates. (B) Heatmap grouping samples (*x*-axis) by control (light yellow) and exposed (gold) corals and H2A peptides (*y*-axis) by *z*-score (complete comparison) of the QC1-normalized area values with dendrograms displayed using Euclidean distance.

**Figure 4.**
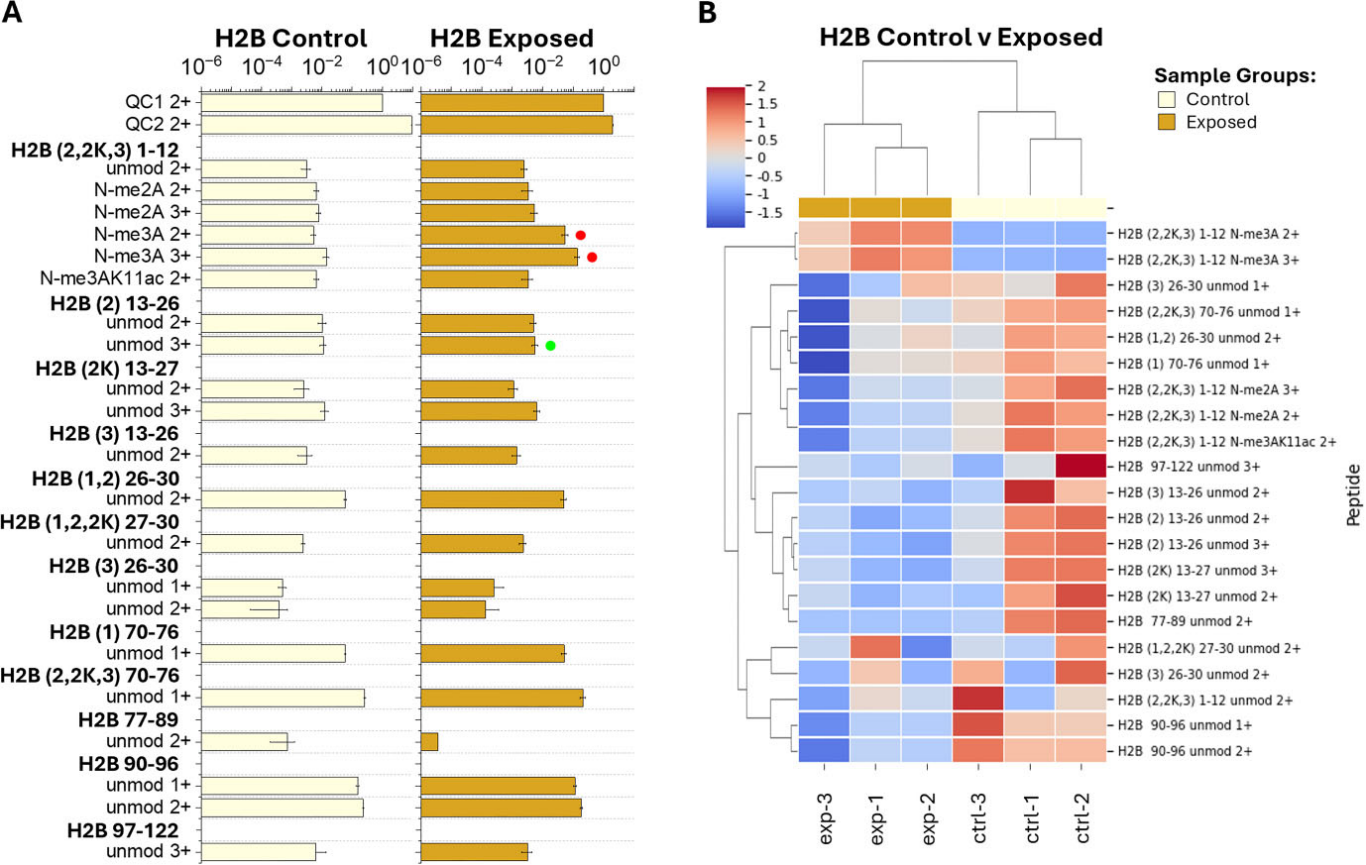
(A) Detected H2B bottom-up peptide areas normalized to QC1 from control and exposed coral samples with up- and downregulated peptide features distinguished by red and green dots, respectively. Bottom-up peptide feature bar plots shown were created from the QC1-normalized area means (bar) and standard deviations (error) across biological replicates. (B) Heatmap grouping samples (*x*-axis) by control (light yellow) and exposed (gold) corals and H2B peptides (*y*-axis) by *z*-score (complete comparison) of the QC1-normalized area values with dendrograms displayed using Euclidean distance.

A target list based on H4, H2A, and H2B variants detected in *A. cervicornis* coral (H4, H4.S, H2A, H2A.A, H2B-1, H2B-2, H2B-2K, and H2B-3; https://doi.org/10.34703/gzx1-9v95/MM9SHA) [42] was used for the verification of the high-sensitivity nLC-TIMS-ddaPASEF-ToF MS/MS method as applied to the analysis of coral response to heat stress exposure (e.g. control and heat-exposed). Inspection of the LC-TIMS-MS/MS analysis resulted in the observation of 84 molecular targets (out of 346 possible molecular targets) corresponding to 8 H4, 7 H2A, and 12 H2B peptides with varying PTMs (Table S1 and Figs 2–4 and Figs S1–S6). Noteworthy is the high reproducibility, sensitivity, and depth of the analysis, which allows the detection of low-abundant peptides and PTMs across six orders of magnitude.

Inspection of the PTM profiles showed correlation with heat exposure across the samples. The number of acetylations on peptide H4 4–17 rose in exposed samples (up to 3ac) versus control samples (up to 1ac). In control samples, the H4 4–17 peptide is only observed in the unmodified and 1ac form (e.g. K8ac 2+ and 3+, K12ac 2+ and 3+, and K16ac 2+ and 3+). However, exposed samples show an increase up to 2 and 3ac, which were below the LOD in the control samples (Figs 1C and 2A and Figs S1 and S2). In both sample groups, H4 4–17 acetylations were observed at K8ac (2+ and 3+), K12ac (2+ and 3+), and K16ac (2+ and 3+). Of the H4 4–17 1ac peptides, K12ac 3+ and K16ac 2+ and 3+ were found to be reduced in the exposed corals, likely due to the increase of peptides with additional acetylations (2–3ac, Figs 1C and 2A, Figs S1 and S2, and Table S2). H4 4–17 peptides with 2ac (K5acK8ac 2+, K5acK12ac 2+, K5acK16ac 2+, K8acK12ac 2+, K8acK16ac 2+, and K12acK16ac 2+) or 3ac (K5acK8acK12ac 2+, K5acK8acK16ac 2+, K5acK12acK16ac 2+, and K8acK12acK16ac 2+) were only observed in the exposed coral samples (Fig. 2A and Figs S1 and S2). All 2 and 3ac H4 4–17 peptides were enriched in the exposed coral samples, compared to the control samples (Figs 1C and 2A, Figs S1 and S2, and Table S2). The H4 20–23 peptide was observed in the unmodified, K20me_1_, K20me_2_, and K20ac forms (Fig. 2A and S1 and S2). The H4 20–23 K20me_2_ 1+ peptide feature was reduced in the exposed coral samples (Fig. 1C and Table S2). All other H4 peptide features (H4 24–35, 40–45, 46–55, 68–78, 79–92, 96–102, and H4.S 79–92) were observed in the unmodified form (Figs S1 and S2).

The H2A 4–16 peptide is observed in both sample groups in the 1ac (K5ac, K7ac, K9ac, K12ac, and K14ac) and 2ac (K5acK7ac, K5acK9ac, K5acK12ac, K7acK9ac, K7acK12ac, K7ac, K14ac, K9acK12ac, K9acK14ac, and K12acK14ac) forms (Figs 3A and Figs S3 and S4). However, the exposed samples show a decrease of 1ac peptide features (K5ac 3+, K7ac 3+, K9ac 3+, K12ac 3+, and K14ac 2+ and 3+), primarily in the 3+ charge state forms (Figs 1C and 3A, Figs S3 and S4, and Table S2). Similarly, H2A 71–76 K74ac (1+ and 2+) was reduced in exposed samples compared to control samples (Fig. 1C and Table S2). All other H2A peptide features (H2A 20–28, 29–34, 35–41, 42–70, and 81–87) were observed in the unmodified form (Figs 3A and Figs S3 and S4). The H2B (2,2K,3) 1–12 peptide was observed in the unmodified, N-me_2_A, and N-me_3_A forms in both sample groups (Figs 4A and Figs S5 and S6). In the exposed samples, an increase of H2B (2,2K,3) 1–12 N-me_3_A 2+ and 3+ peptide features was observed compared to the control group (Figs 1C and 4A, Figs S5 and S6, and Table S2). All other H2B peptide features [H2B [2] 13–26, (2K) 13–27, [3] 13–26, [1, 2] 26–30, (1,2,2K) 27–30, [3] 26–30, [1] 70–76, (2,2K,3) 70–76, 77–89, 90–96, 97–722] were observed in the unmodified form (Figs 4A and Figs S5 and S6).

Clustering of the samples by heat exposure treatment indicates a clear distinction between control and exposed sample responses to heat stress for H4 (Fig. 2B), H2A (Fig. 3B), and H2B (Fig. 4B) PTMs. The sample comparison between treatments shows tight clustering among most similar PTMs [e.g. H4 4–17 1ac, 2ac, and 3ac, Fig. 2B; H2A 4–16 1ac and 2ac, Fig. 3B; H2B (2,2K,3) 1–12 N-me_2_A and N-me_3_A, Fig. 4B]. Additionally, clustering is observed between some peptide features with similar PTMs and charge states (i.e. H4 4–17 K12ac 3+ and K16ac 3+, Fig. 2B; H2A 4–16 K5ac 3+ and K7ac 3+, Fig. 3B) as well as some peptides detected at multiple charge states (i.e. H4 20–23 K20me_2_ 1+ and 2+, Fig. 2B; H2A K12ac 2+ and 3+, and H2A K14ac 2+ and 3+, Fig. 3B). These types of observations are expected, as peptides with multiple charge states observed would see similar decreases/increases within each charge state relevant to total peptide abundance.

Understanding changes in the epigenetic marks in corals is critical to advance our interpretation and propose gene regulation mechanisms and pathways. In particular, the elucidation and quantification of histone PTMs requires advanced analytical detection methods due to the ubiquitously high isomeric content and dynamic range. Online nLC-TIMS-ddaPASEF-ToF MS/MS can be efficiently used for the bottom-up analysis of derivatized histone peptides when applied to the *A. cervicornis* Caribbean staghorn coral.

A high diversity of core histone PTMs was observed in response to heat stress. The inspection of *A. cervicornis* H4, H4.S, H2A, H2A.A, H2B-1, H2B-2, H2B-2K, and H2B-3 showed the presence of varying numbers of acetylation (0–3ac) and methylation (me_0-3_), mostly in the tail positions. The analysis was aided by the ability to isolate mass- and mobility-selected precursors for increased peptide assignment by increasing signal-to-noise and reducing the number of interfering ions [38, 41], despite the high diversity of PTMs among samples. When compared, increases in H4 4–17 with 2ac and 3ac, and decreases in H4 4–17 K12ac, K16ac, H4 K20me_2_, and H2A K5ac, K7ac, K9ac, K12ac, K14ac, and K74ac were observed from corals exposed to heat stress (exposed) versus those held at ambient temperature (control). While a direct correlation between these PTMs and chromatin remodelling remains to be elucidated, previous reports have shown their involvement in gene transcription [43, 44], DNA replication [45], and DNA damage repair [46]. This work introduced a new analytical workflow capable of characterizing chromatin composition in corals, providing the community with tools for future longitudinal studies and the evaluation of responses of other non-model organisms to environmental change.

## Supplementary Material

dvaf017_Supplemental_File

## Data Availability

Bottom-up nLC-TIMS-ddaPASEF-ToF MS/MS raw data for all ambient control and heat-exposed *A. cervicornis* samples are freely accessible *via* the FIU Research Data Repository at https://doi.org/10.34703/gzx1-9v95/3MF7D3. Top-down LC-MS, nESI-ToF, and nESI-q-ECD-ToF MS/MS raw data for *A. cervicornis* histone H4 variant sequences are freely accessible *via* the FIU Research Data Repository at https://doi.org/10.34703/gzx1-9v95/MM9SHA.
