# Supplementary material for: Mapping heat stress-induced core histone post-translational modifications in Acropora cervicornis
Source: Environ Epigenet. 2025 May 29;11(1):dvaf017. doi: 10.1093/eep/dvaf017 (PMC12418939; doi:10.1093/eep/dvaf017)
Supplement: dvaf017_Supplemental_File [file dvaf017_supplemental_file.pdf]

**Supplementary Information**

# Mapping Heat Stress-Induced Core Histone Post-Translational Modifications in *Acropora cervicornis*.

Cassandra N. Fuller[1], Sabrina Mansoor[2], Santiago J. Guzman[1], Lilian Valadares Tose[1], Serena Hackerott[2,3], Javier Rodriguez-Casariego[2,4], Jose M. Eirin-Lopez[2*], Francisco Fernandez-Lima[1,5*]

[1]*Department of Chemistry and Biochemistry, Florida International University, Miami, FL 33199, USA.*

[2]*Environmental Epigenetics Laboratory, Institute of Environment, Florida International University, Miami, FL 33199, USA.*

[3]*College of Earth, Ocean, and Environment, School of Marine Science and Policy, University of Delaware, Lewes, DE 19958, USA*

[4]*Department of Marine Biology and Ecology, Rosenstiel School, University of Miami, Miami, FL 33124, USA.*

[5]*Biomolecular Sciences Institute, Florida International University, Miami, FL 33199, USA.*

## Contents

Table S2. Table of the upregulated and downregulated core histone peptide features in coral samples after heat exposure. .......... 3

Figure S1. Raw H4 peptide feature areas for control and exposed coral samples. Limit of detection shown by the red dashed line. .......... 4

Figure S2. H4 peptide feature areas normalized to QC1 for control and exposed coral samples. Limit of detection shown by the red dashed line. .......... 5

Figure S3. Raw H2A peptide feature areas for control and exposed coral samples. Limit of detection shown by the red dashed line. .......... 6

Figure S4. H2A peptide feature areas normalized to QC1 for control and exposed coral samples. Limit of detection shown by the red dashed line. .......... 8

Figure S5. Raw H2B peptide feature areas for control and exposed coral samples. Limit of detection shown by the red dashed line. .......... 10

Figure S6. H2B peptide feature areas normalized to QC1 for control and exposed coral samples. Limit of detection shown by the red dashed line. ..........11

Figure S7. Estimation of the method limit of detection based on peptide pr-AK(ac)AK(pr)TR ($828.4938^{1+}$) from a bovine histone pulldown. .................................................................. 12

**Table S2.** Table of the upregulated and downregulated core histone peptide features in coral samples after heat exposure.

| Regulation of Peptide Features Post Heat Exposure | |
|---|---|
| **Upregulated** | **Downregulated** |
| H4 4-17 K5acK8ac 2+ | H4 4-17 K12ac 3+ |
| H4 4-17 K5acK12ac 2+ | H4 4-17 K16ac 2+ |
| H4 4-17 K5acK16ac 2+ | H4 4-17 K16ac 3+ |
| H4 4-17 K8acK12ac 2+ | H4 20-23 K20me2 1+ |
| H4 4-17 K8acK16ac 2+ | H2A 4-16 K5ac 3+ |
| H4 4-17 K12acK16ac 2+ | H2A 4-16 K7ac 3+ |
| H4 4-17 K5acK8acK12ac 2+ | H2A 4-16 K9ac 3+ |
| H4 4-17 K5acK8acK16ac 2+ | H2A 4-16 K12ac 3+ |
| H4 4-17 K5acK12acK16ac 2+ | H2A 4-16 K14ac 2+ |
| H4 4-17 K8acK12acK16ac 2+ | H2A 4-16 K14ac 3+ |
| H2B (2,2K,3) 1-12 N-me3A 2+ | H2A 71-76 K74ac 1+ |
| H2B (2,2K,3) 1-12 N-me3A 3+ | H2A 71-76 K74ac 2+ |

**Figure S1.** Raw H4 peptide feature areas for control and exposed coral samples. Limit of detection shown by the red dashed line.

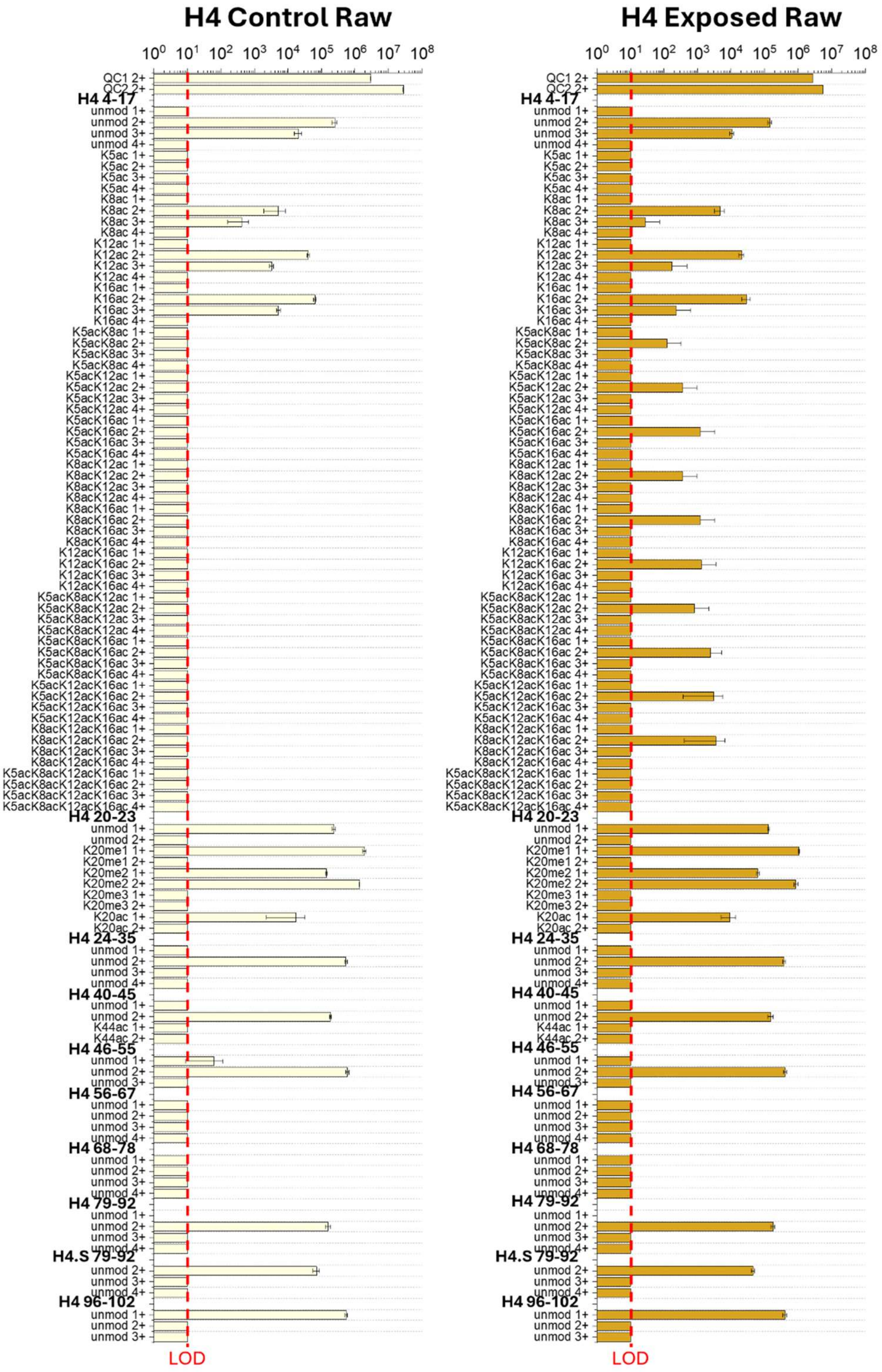

**Figure S2.** H4 peptide feature areas normalized to QC1 for control and exposed coral samples. Limit of detection shown by the red dashed line.

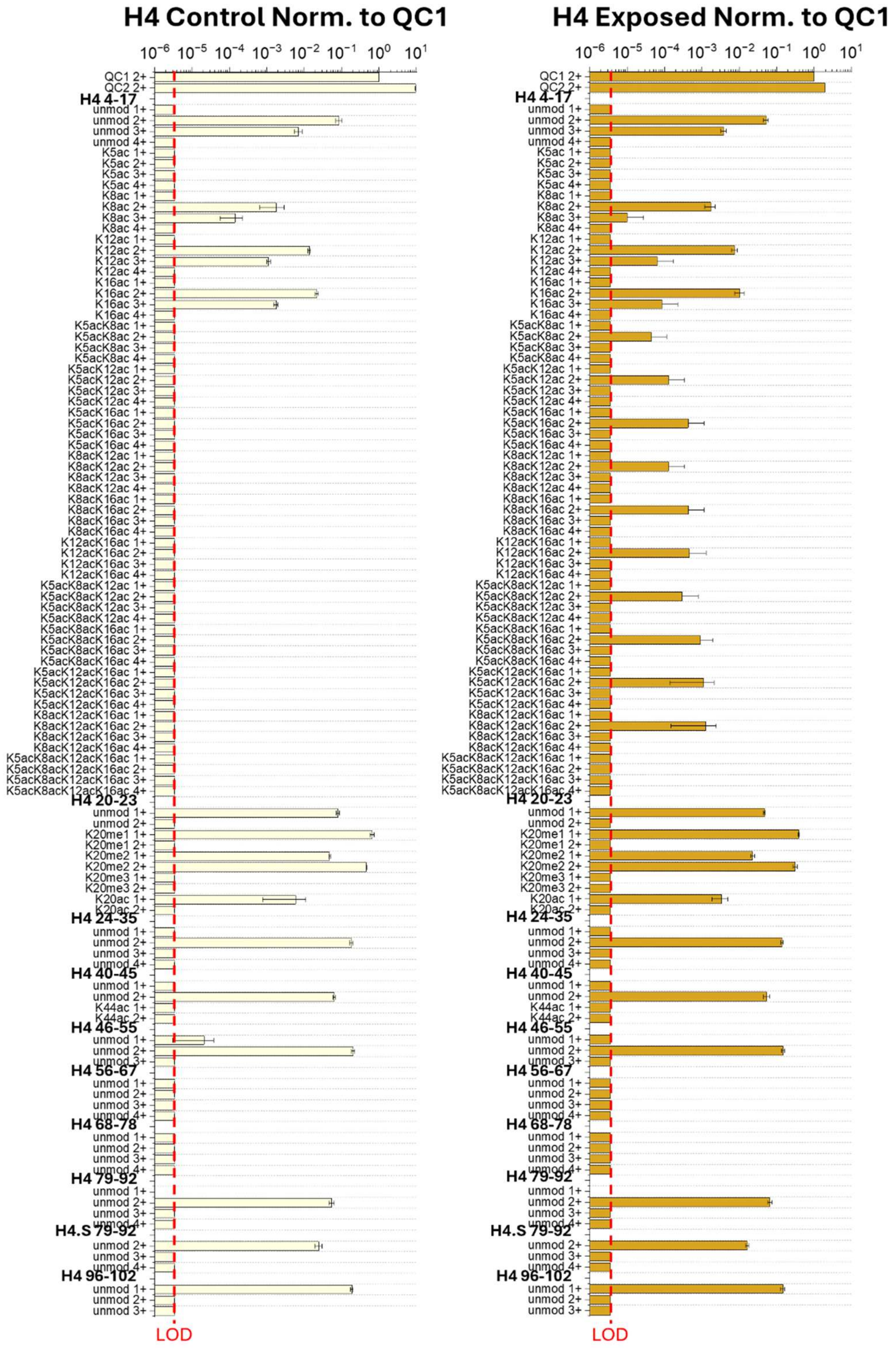

**Figure S3.** Raw H2A peptide feature areas for control and exposed coral samples. Limit of detection shown by the red dashed line.

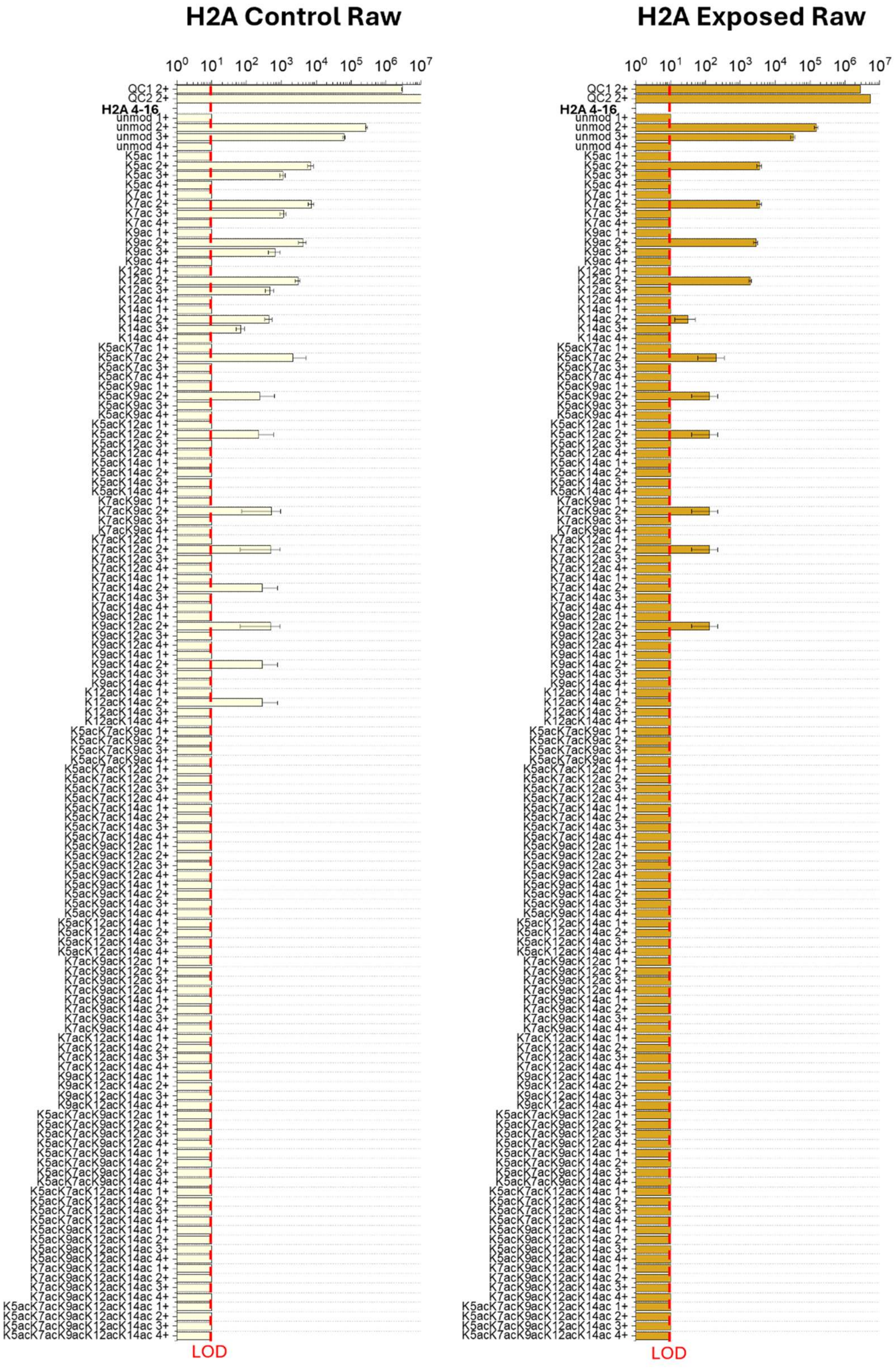

**Figure S3 continued.** Raw H2A peptide feature areas for control and exposed coral samples. Limit of detection shown by the red dashed line.

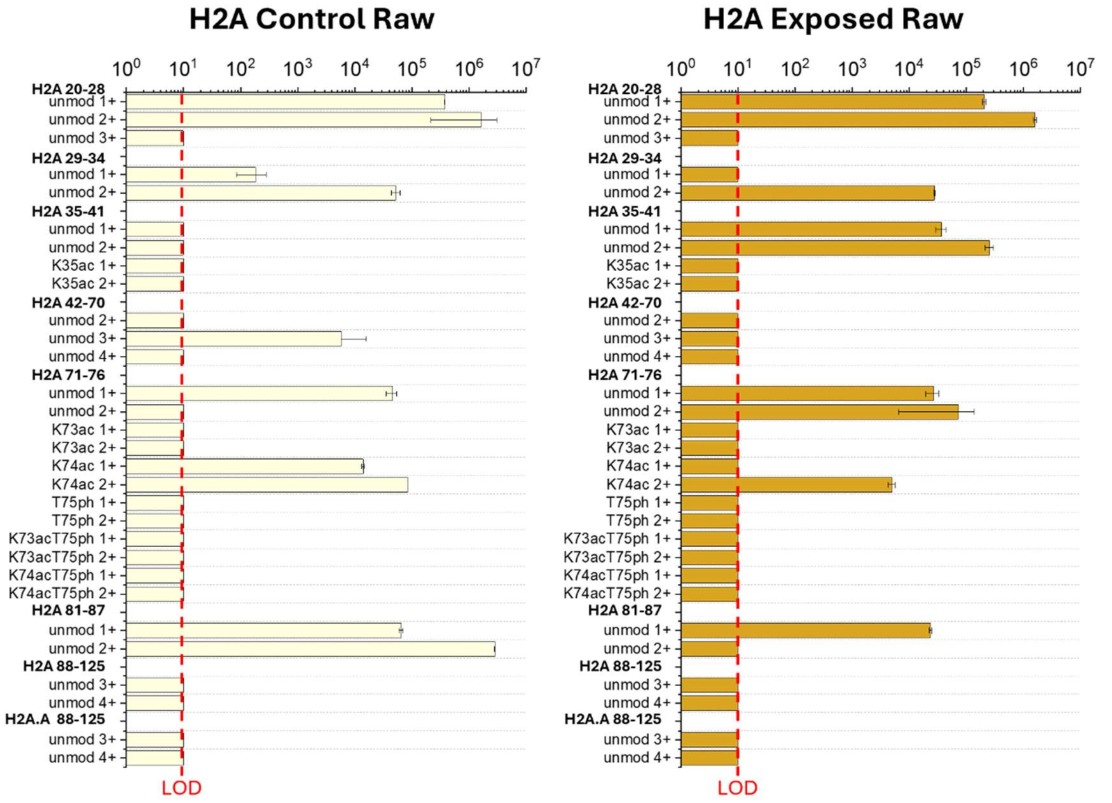

**Figure S4.** H2A peptide feature areas normalized to QC1 for control and exposed coral samples. Limit of detection shown by the red dashed line.

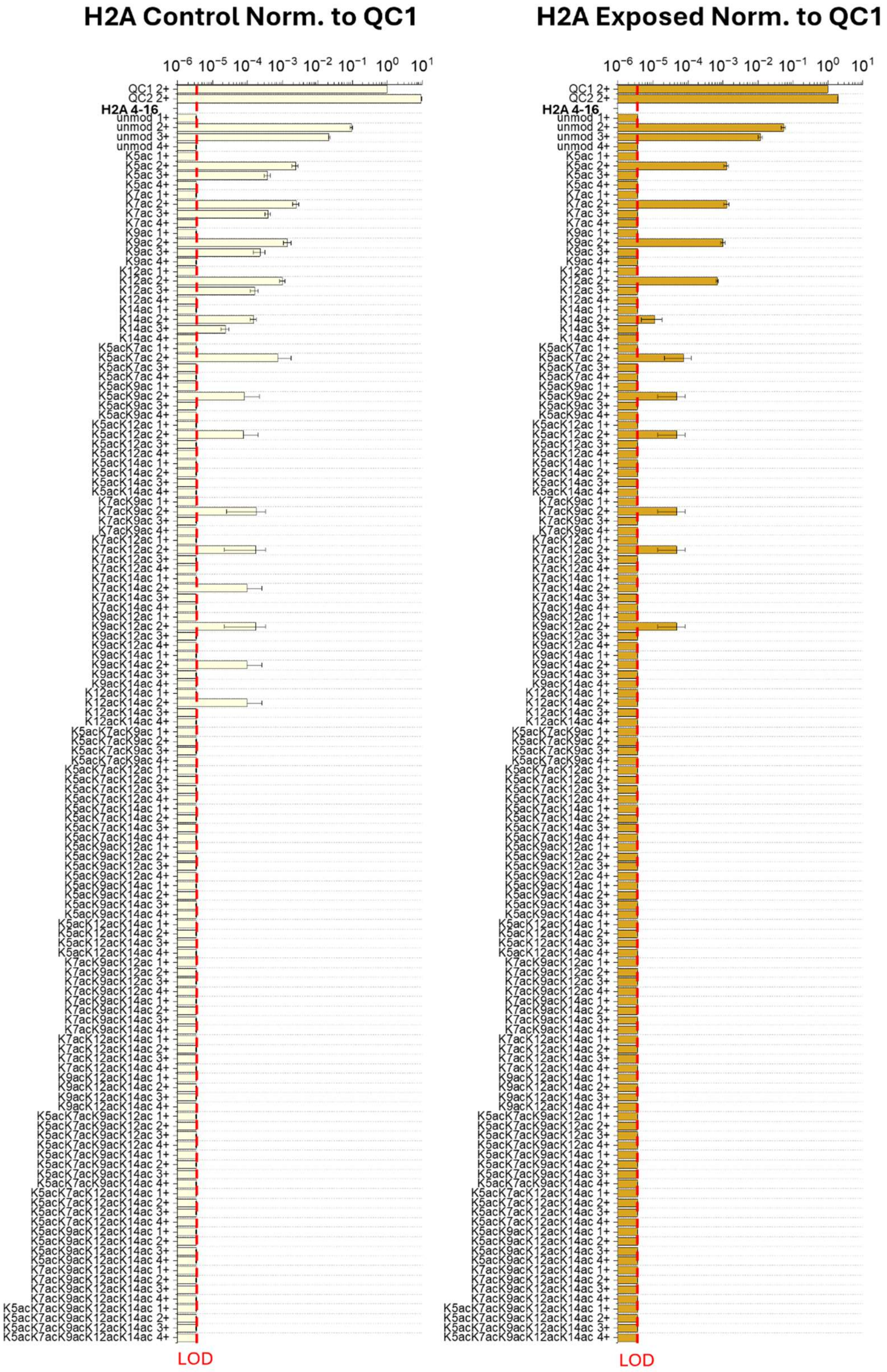

**Figure S4 continued.** H2A peptide feature areas normalized to QC1 for control and exposed coral samples. Limit of detection shown by the red dashed line.

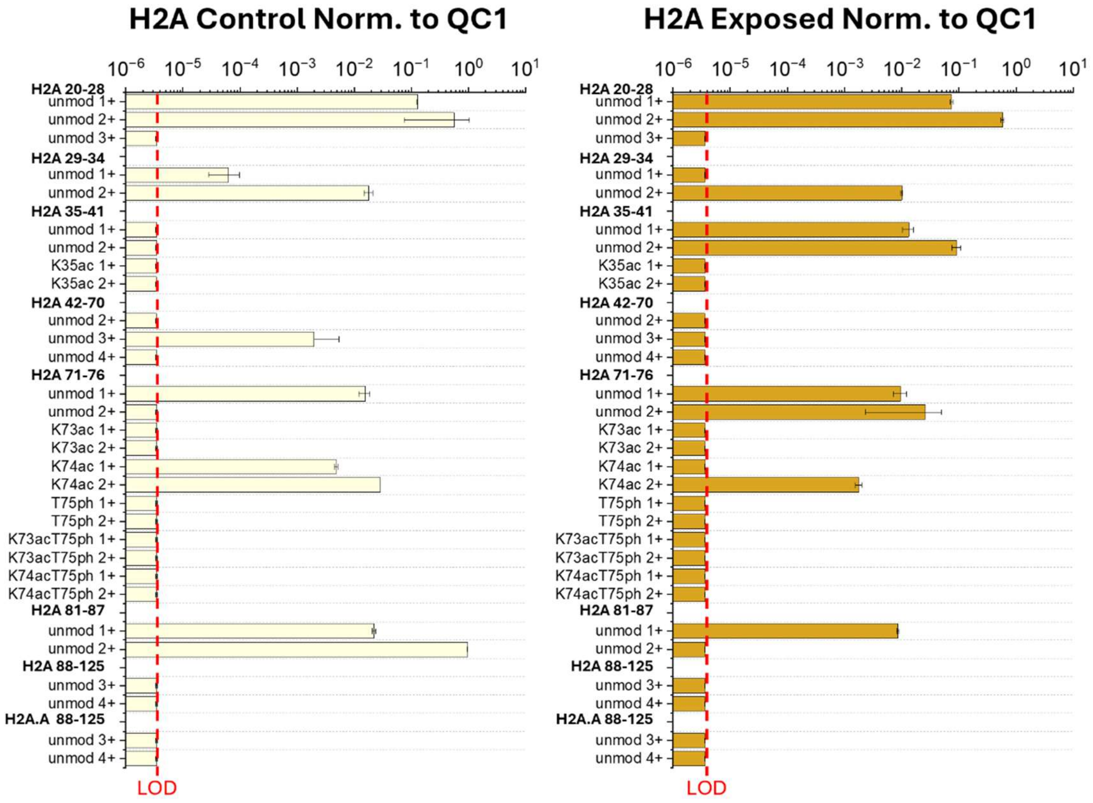

**Figure S5.** Raw H2B peptide feature areas for control and exposed coral samples. Limit of detection shown by the red dashed line.

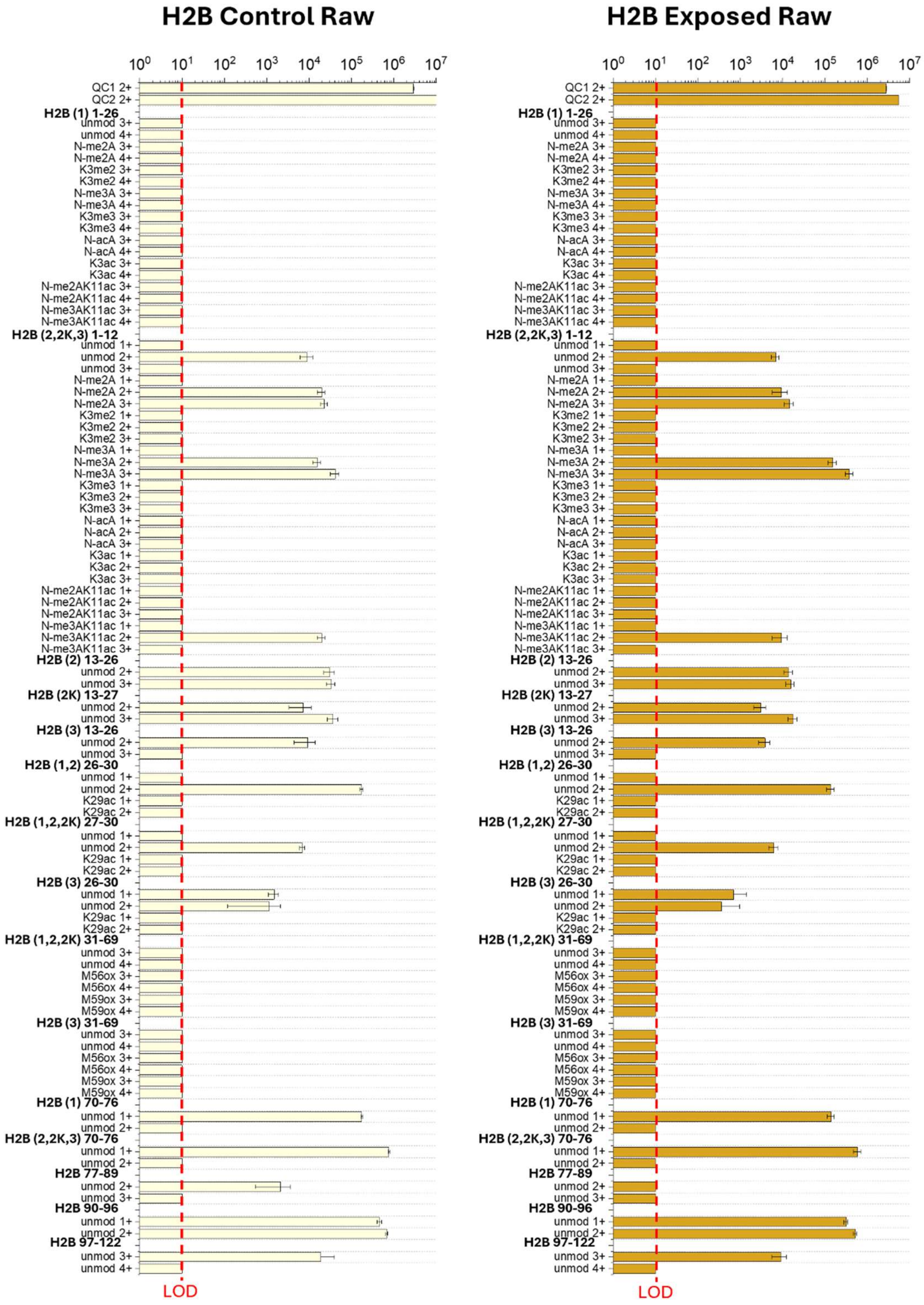

**Figure S6.** H2B peptide feature areas normalized to QC1 for control and exposed coral samples. Limit of detection shown by the red dashed line.

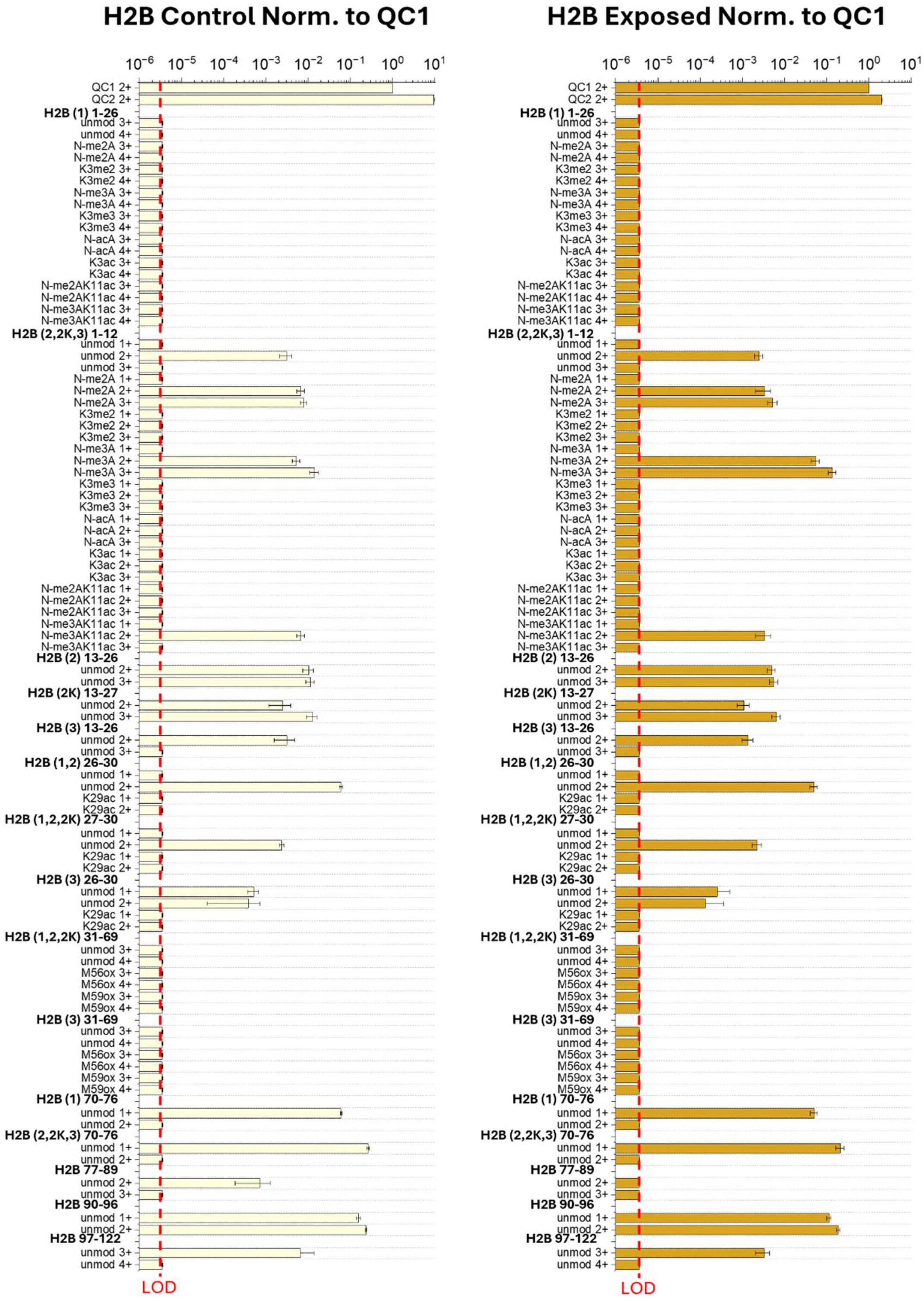

**Figure S7.** Estimation of the method limit of detection based on peptide pr-AK(ac)AK(pr)TR (828.4938[1+]) from a bovine histone pulldown.

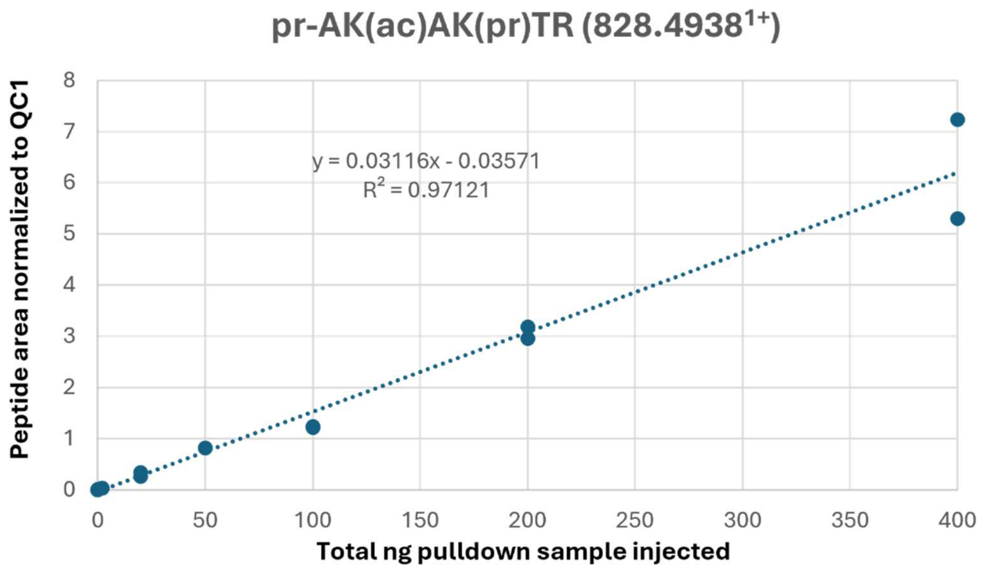


| pr-AK(ac)AK(pr)TR ($828.4938^{1+}$) | |
|---|---|
| **LOD signal** | 9.931 |
| **LOQ signal** | 49.656 |
